# ﻿*Liliumhuanglongense* (Liliaceae): a newly-discovered species in north-western Sichuan, China

**DOI:** 10.3897/phytokeys.252.135155

**Published:** 2025-02-04

**Authors:** Ting Wang, Yumei Yuan, Ting-Hong Zhou, Yundong Gao

**Affiliations:** 1 CAS Key Laboratory of Mountain Ecological Restoration and Bioresource Utilization & Ecological Restoration and Biodiversity Conservation Key Laboratory of Sichuan Province, Chengdu Institute of Biology, Chinese Academy of Sciences, Chengdu 610213, China Chengdu Institute of Biology, Chinese Academy of Sciences Chengdu China; 2 University of Chinese Academy of Sciences, Beijing 100049, China University of Chinese Academy of Sciences Beijing China; 3 Administrative Bureau of the Huanglong Scenic and Historic Interest Area, Songpan 623300, China Administrative Bureau of the Huanglong Scenic and Historic Interest Area Songpan China

**Keywords:** Liliaceae, *Liliumhuanglongense*, Lophophorum-clade, new species, section *Lophophora*

## Abstract

In this study, we describe *Liliumhuanglongense*, a newly-discovered lily species identified following extensive surveys in an undeveloped area of the Huanglong National Nature Reserve in Sichuan, China. This region, located in the Hengduan Mountains of south-western China, is recognised as one of the world’s prominent biodiversity hotspots, providing diverse habitats for a wide range of plant species. Morphologically, *L.huanglongense* resembles *Liliumfargesii* Franch., which is distributed in central China, as well as other tepal-recurved members of the section Lophophora (Bureau & Franch.) F. T. Wang & Ts. Tang. This section comprises dwarf lilies predominantly found in the alpine scrub of the Hengduan Mountains, extending westwards into the Himalayas. Molecular phylogenetic analyses using both nuclear ITS and chloroplast genomes confirm the independent status of the new species and its placement within the section Lophophora. The identification of this new species helps to fill the distribution gap between broad-leaved forest and alpine scrub species within the section, thereby enhancing our understanding of the diversity and distribution history of *Lophophora*.

## ﻿Introduction

*Lilium*, a genus in the tribe Lilieae of the family Liliaceae, comprises herbaceous, bulbous plants with scaled bulbs, dorsifixed anthers and loculicidal capsules (Peruzzi 2016). With approximately 123 recognised species (POWO 2024), the genus is widely distributed across the Northern Hemisphere in Asia, Europe and North America (Liang and Tamura 2000). China, in particular, hosts approximately 55 distinct species according to the latest flora records (Liang 1995; Liang and Tamura 2000), with key distribution areas including northeast, central and south-western China. Amongst these regions, south-western China stands out as a hotspot for the diversity of wild lilies due to its mountainous environment (Gao et al. 2013b, 2015). These mountain ranges and deep valleys constitute an intricate topography, which provides unique habitats for various species.

The Daba Mountains and the Qinling Mountains of central China, as well as the Hengduan Mountains and the Himalayas, form a series of mountain ranges from central to western China, together harbouring the greatest number of lilies in the world (Yundong Gao, unpublished data). Furthermore, the rugged terrain and sparse population in mountainous regions have constrained previous explorations, indicating the potential presence of undiscovered species in these areas. Investigating plant groups within these continuous mountain ranges would enhance our understanding of species diversification and dispersal history amongst the selected plant species, thereby contributing significantly to our overall knowledge of biodiversity.

**Figure 1.**
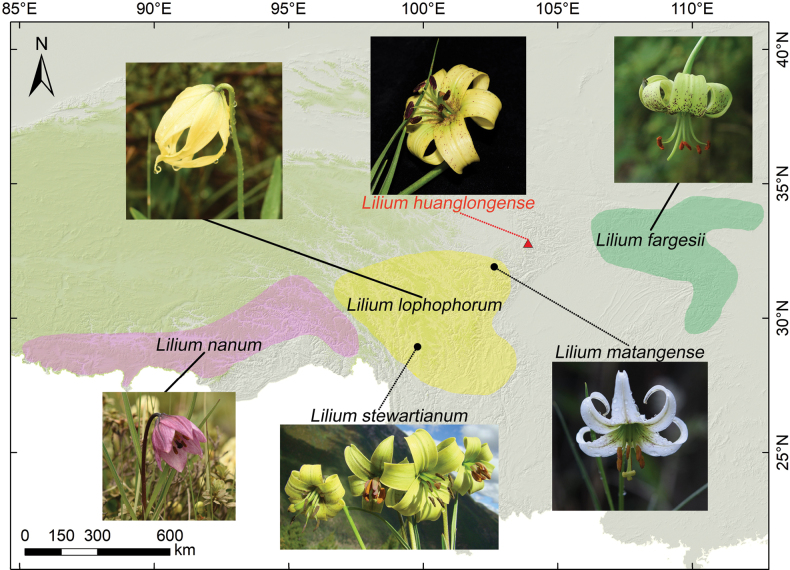
Morphological characteristics and geographic distribution of *Liliumhuanglongense* and related species. *Liliumhuanglongense* fills the geographic gap west of the Qinling Mountains and at the confluence of the Hengduan Mountains.

Our prior investigation elucidated the taxonomic classification of the genus *Lilium* (Gao et al. 2012, 2013b, 2015; Gao and Gao 2016; Yuan and Gao 2024). Specifically, the Lophophorum-clade *sensu*Gao et al. (2013b) was identified, which comprises both the campanulate-flowered *L.oxypetalum* Baker, *L.lophophorum* (Bureau & Franch.) Franch., *L.nanum* Klotzsch, as well as the tepal-recurved *L.fargesii* Franch., *L.stewartianum* Balf. f. & W.W. Sm. and *L.matangense* J.M. Xu that span mountainous regions from central China (broad-leaved forests) to the Himalayas (alpine scrub and meadows). This clade is analogous to the subgeneric section Lophophora (Bureau & Franch.) F. T. Wang & Ts. Tang, as refined by Watanabe et al. (2021) in their recent work. Most members of this clade, which exhibit recurved perianths, have a limited and sporadic distribution. For example, *L.fargesii* exhibits a notable widespread distribution at mid-elevation areas (second step) in China (mainly in Qinling and Daba Mountains), while *L.matangense* is found further westwards at higher altitudes of over 3000 m, with narrow distribution and very small population sizes and therefore is of conservation value. Additionally, *L.stewartianum* is distributed at an altitude of about 3500 m in the southern Hengduan Mountains (Fig. 1).

With advancements in molecular phylogenetics, the monophyly of the Lophophorum-clade has been confirmed (Gao et al. 2013b; Watanabe et al. 2021); however, our understanding of its composition and evolutionary history remains incomplete. Moreover, the distribution of the entire Lophophorum-clade is disjunctive (Fig. 1), particularly between the northern Hengduan Mountains and the western Qinling Mountains, creating a “gap” where members of this clade have not been previously documented. This relic distribution pattern may suggest the presence of additional undiscovered taxa or those that are already extinct. In recent years, meticulous sampling and analysis of this group have yielded several novel findings. This paper highlights one such discovery: the putative new species *L.huanglongense*, identified by rangers in the Huanglong National Nature Reserve. This discovery can be partially attributed to the increased attention that the Chinese government has directed towards nature reserves and it represents a significant advancement in our understanding of this clade.

Currently, we aim to clarify the status and phylogenetic position of the putative new species by comparing its morphology with that of the most morphologically similar species, in addition to conducting molecular phylogenetic analyses utilising both nuclear markers and chloroplast genomes. Furthermore, the analysis of the morphological and genetic distinctiveness of *L.huanglongense* is expected to offer additional insights into the Lophophorum-clade by addressing the geographic distribution gap observed amongst its members.

## ﻿Materials and methods

### ﻿Field sampling

Leaf materials of the new species were collected from the Huanglong National Nature Reserve and temporarily preserved in silica gel for DNA extraction. During fieldwork, we captured many photographs of the individuals and collected three complete specimens for conservation purposes. These images and specimens were used for subsequent measurements and descriptions. The voucher specimens have been deposited in the Herbarium of the Chengdu Institute of Biology (**CDBI**).

### ﻿Morphological analysis

This study is grounded in an analysis of herbarium specimens, digital specimen images, field observations and relevant literature. We conducted a comprehensive literature review of pertinent taxa using online databases such as Tropicos (https://tropicos.org/) and the Biodiversity Heritage Library (BHL, https://www.biodiversitylibrary.org/), focusing on *Liliumoxypetalum* Baker (Baker 1874), *L.lophophorum* (Bureau & Franch.) Franch. (Franchet 1898), *L.nanum* Klotzsch (Klotzsch and Garcke 1862), *L.fargesii* Franch. (Franchet 1892), *L.stewartianum* Balf. f. & W.W. Sm. (Smith 1923) and *L.matangense* J.M. Xu (Xu 1985).

Specimens were meticulously examined through visits to the CDBI, IBSC, KUN, PE, SZ and WUK Herbaria (acronyms according to Thiers (2024), same below) and by accessing digital images from virtual herbarium platforms, including the China Virtual Herbarium (https://www.cvh.ac.cn/), the Kew Herbarium Catalogue (http://apps.kew.org/herbcat/gotoHomePage.do) and JSTOR Global Plants (https://plants.jstor.org/), as well as online images from herbaria B, E, GH, K and P. This approach aimed to facilitate a comparative analysis of morphological characters based on a substantial number of specimens. Morphological traits were selected, based on taxonomically significant features detailed in the “Flora of China” (Liang and Tamura 2000), including bulbs, stems, leaves and flowers. Specifically, the new species was morphologically compared to the tepal-recurved members of the Lophophorum-clade, namely *L.fargesii*, *L.stewartianum* and *L.matangense*. For comparative analysis, the dimensions of the bulbs, stems, leaves and floral organs were measured from both specimen images and photographs of fresh plants, utilising MATO (Liu et al. 2023) and PS software (Suppl. material 1: table S1). The Extent of Occurrence (EOO) and Area of Occupancy (AOO) were calculated using the GeoCAT software (Bachman et al. 2011).

### ﻿Molecular phylogeny inference

Genomic DNA was extracted from silica-gel dried leaves using a modified cetyltrimethylammonium bromide (CTAB) method (Allen et al. 2006). Paired-end sequencing libraries were then constructed with insert sizes of approximately 350 bp, followed by sequencing on the DNBSEQ-T7 platform (Beijing Genomics Institute, BGI), with the depth of about 0.1 ~ 0.2 × (10G pair ending reads). About 13 Gb of raw data were filtered by fastp v.0.23.2 (Chen et al. 2018). The Internal Transcribed Spacer (ITS1, 5.8S and ITS2) and chloroplast genome of new species were then assembled using GetOrganelle v.1.7.6.1 (Jin et al. 2020) with default parameters. Chloroplast genomes were annotated and manual corrections were made using Geneious Prime v.2023.1.2 (Biomatters Ltd. Auckland, New Zealand), based on the plastome of *Liliumfargesii* (NC_033908.1).

To deduce the phylogenetic position of the putative new species, we combined newly-generated DNA sequences and published sequences, including thirty-two ITS and twenty-eight cp genome from NCBI (https://www.ncbi.nlm.nih.gov/), to infer phylogenetic relationships, selecting the entire Lophophorum-clade species and 2–3 representative from closely-related clades (Suppl. material 1: table S2) based on previous studies (Gao et al. 2013a; Yuan and Gao 2024). Outgroups included four species of *Fritillaria* and *Cardiocrinum* (Suppl. material 1: table S2).

We utilised the online platform (https://ngphylogeny.fr/, Lemoine et al. (2019)) to construct Maximum Likelihood (ML) phylogenetic trees based on complete plastid sequences. The sequences were analysed through the Advanced Workflow, employing the PhyML + SMS/OneClick method. Detailed workflows for MAFFT, BMGE and PhyML + SMS (Maximum Likelihood-Based Phylogenetic Tree Inference with Intelligent Model Selection) are provided in the Methods section of Lemoine et al. (2019). Bootstrap analysis (FBP + TBE) was conducted with 1000 replicates, while all other parameters were kept at their default settings. ITS sequences were processed using PhyloSuite v.1.2.2 (Zhang et al. 2020). A total of 34 sequences were aligned in batches with MAFFT v.7.313 (Katoh and Standley 2013) using the ‘--auto’ strategy in normal alignment mode. The resultant files were subjected to additional manual corrections using MEGA v.11.0 (Tamura et al. 2021). Subsequent analyses were performed in PhyloSuite, where ambiguous sites and gaps were removed using Gblocks (Talavera and Castresana 2007). The sequences were then concatenated into a single alignment and converted into Nexus format files.

ModelFinder (Kalyaanamoorthy et al. 2017) was used for the selection of the most appropriate evolutionary model. Based on the Akaike Information Criterion, GTR + F + G4 was chosen as the optimal model of nucleotide evolution. Bayesian phylogenies were inferred using MrBayes 3.2.6 (Ronquist et al. 2012) under partition model (2 parallel runs, 10,000,000 generations), in which the initial 25% of sampled data were discarded as burn-in. The construction of the ITS Maximum Likelihood (ML) tree was performed using PhyloSuite, with the sequence file generated through MAFFT and Gblocks. The file was then processed using the A La Carte option on the online tree-building platform to execute the PhyML analysis. Bootstrap analysis was conducted with 1000 replicates and all other parameters were set to their default values. The generated Maximum Likelihood (ML) and Bayesian Inference (BI) (Suppl. material 2) phylogenetic trees were visualised using iTOL v.6 (https://itol.embl.de, Letunic and Bork (2024)).

## ﻿Results

### ﻿Morphology comparison (Figs 2–4, Table 1)

**Figure 2.**
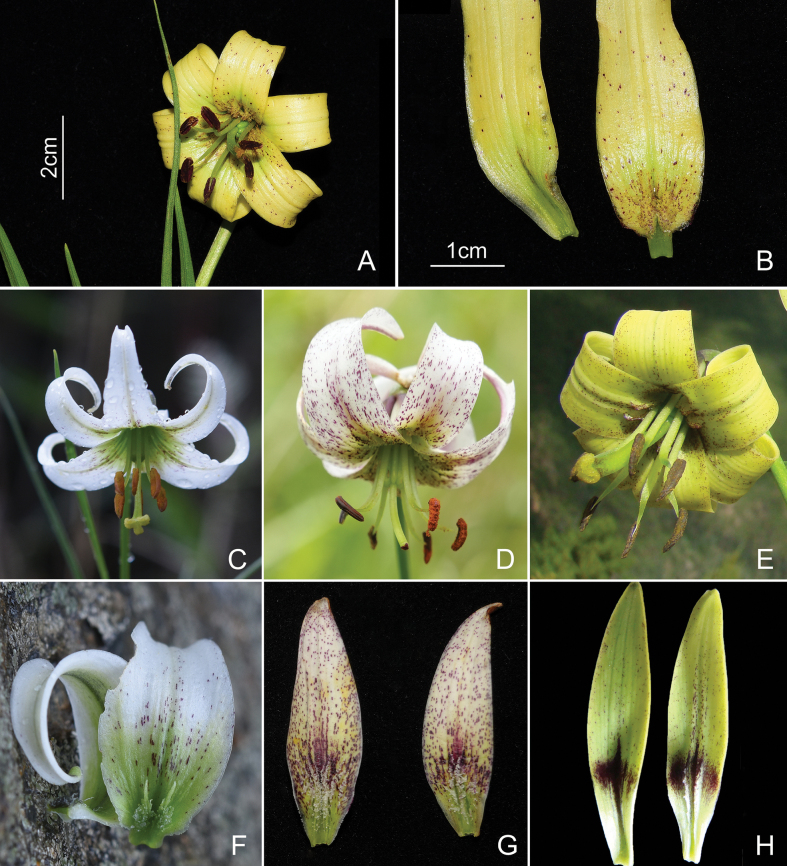
Comparison of floral structures of similar species **A***Liliumhuanglongense* flower **B** tepals of *Liliumhuanglongense* with basal nectaries **C***Liliummatangense* flower **D***Liliumfargesii* flower **E***Liliumstewartianum* flower **F** tepals of *Liliummatangense* with basal nectaries **G** tepals of *Liliumfargesii* with basal nectaries **H** tepals of *Liliumstewartianum* with basal nectaries. Photographed by Yundong Gao.

*Liliumhuanglongense* shares with *L.lophophorum* a pair of marginal ridges along the central groove on the adaxial surface of tepals (Fig. 2B), which has been recognised as the most important character defining this section (Watanabe et al. 2021). This feature also provides morphological evidence supporting the new species’ placement within the Lophophorum-clade. Additionally, the turk’s-cap perigone suggests that *L.huanglongense* is more closely related to tepal-recurved species such as *L.fargesii*.

**Figure 3.**
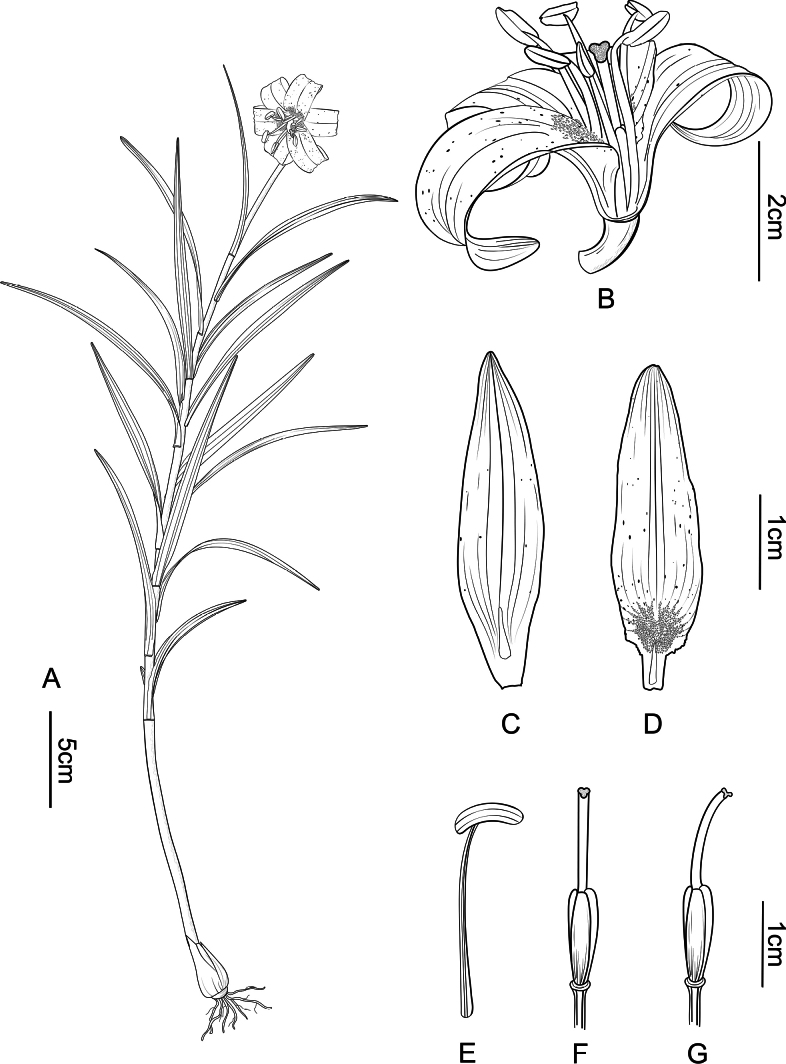
*Liliumhuanglongense* T.Wang & Y.D.Gao, sp. nov. **A** habit **B** dissected flower **C** outer perianth segment **D** inner perianth segment **E** stamen **F** pistil (frontal view) **G** pistil (lateral view). Drawn by T. Wang from the holotype.

While *L.huanglongense* shares reflexed perianth segments with *L.fargesii*, *L.stewartianum* and *L.matangense*, it differs notably in terms of floral organs. Firstly, the flower of the new species is about 3–4 cm in diameter and, when fully expanded, the perianth is nearly in the same plane as the androgynophore (Fig. 2A), whereas in the other species, the androgynophore is exposed to a greater extent (Fig. 2C–E). Secondly, the stigma of *L.huanglongense* is three-lobed without inflation (Fig. 3F, G), whereas that of *L.matangense* is three-lobed with inflation (Fig. 2C).

**Figure 4.**
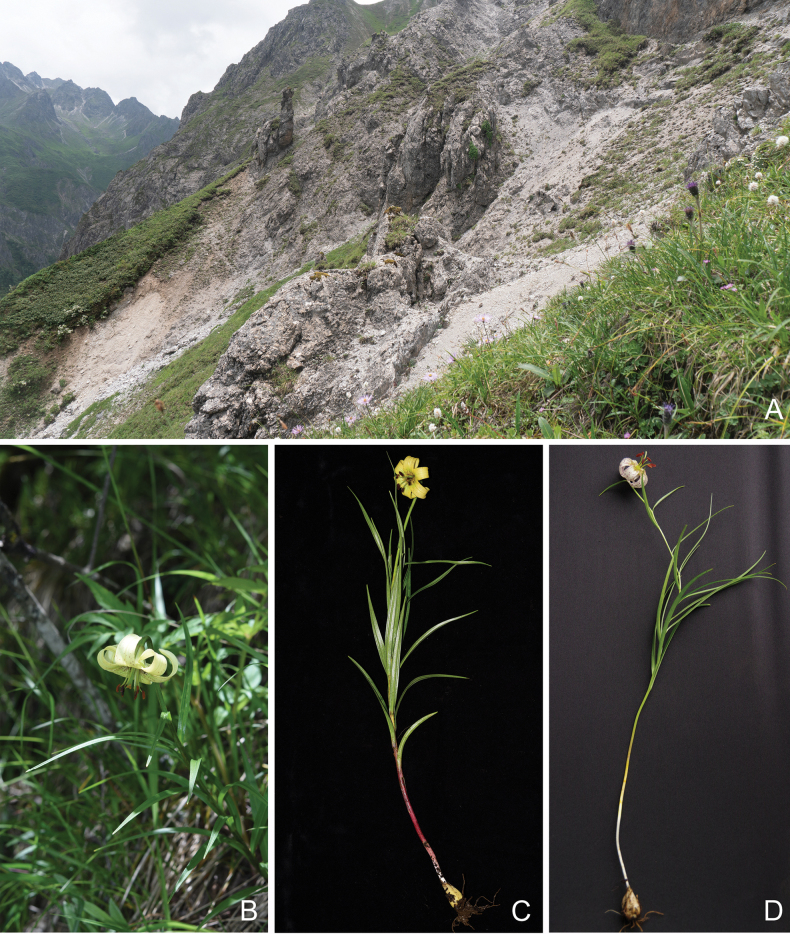
Habitat of *Liliumhuanglongense* and morphological comparison with *Liliumfargesii***A** habitat destroyed by mudslides **B** flowering plant **C** habit of *Liliumhuanglongense* exhibiting a greater abundance of basal leaves, accompanied by wider leaf blades compared to *L.fargesii***D** habit of *Liliumfargesii*. *Liliumhuanglongense*. Photographs were taken by multiple authors of present work.

Furthermore, *L.huanglongense* possesses a greater number of basal leaves compared to *L.fargesii* (Fig. 4C, D). The detailed differences between *L.huanglongense* and the most similar species are listed in Table 1. These morphological differences effectively distinguish the new species from known congeners.

**Table 1.** Morphological comparisons of *Liliumhuanglongense*, *L.fargesii*, *L.stewartianum*, and *L.matangense*.

Characters		*L.huanglongense*	*L.fargesii*	*L.stewartianum*	*L.matangense*
Bulb	colour	yellow	white	yellow	white
	diam.	1.2–1.5 cm	approximately 1.5 cm	approximately 2.0 cm	1.0–1.5 cm
Stem	length	15–40 cm	20–70 cm	20–50 cm.	23–35 cm
Leaves		5–12 × 0.3–0.7 cm	10–14 × 0.2–0.5 cm	2.5–7 × 0.3–0.4 cm	2–2.5 × 0.5–1 cm
Flower	basal colour	yellow	green, pink	greenish to deep yellow	white
	tube length	shorter	shorter	longer	shorter
	stigma	three-lobed without inflation	three-lobed without inflation	three-lobed with inflation	three-lobed with inflation
	nectar glands	nectaries with cristate projections on both surfaces	nectaries with cristate projections on both surfaces	papillose nectaries that form two ridges along the bases of the inner tepals	inner ones with fimbriate projections on both surfaces of nectaries

### ﻿Phylogenetic analyses (Fig. 5)

The analysis was based on molecular data, specifically ITS (ITS1, 5.8S and ITS2) sequences and the complete chloroplast genome. This study utilised two datasets, each including two individuals of the new species. The new species has an ITS sequences with 624 base pairs (bp) in length with a GC content of 61.5%, whereas the chloroplast genome was 152,597 bp long with a GC content of 37.0%. The chloroplast genome comprises double-stranded circular DNA and exhibits a characteristic quadripartite structure, including a large single-copy (LSC) region spanning 81,965 bp, a small single-copy (SSC) region of 17,496 bp and two inverted repeat (IR) regions, each measuring 26,568 bp. We utilised 34 ITS sequences, with lengths ranging from 610 bp to 633 bp prior to alignment and, after alignment correction, the sequence lengths were 641 bp with 223 variable sites and 411 conserved sites. In addition, we analysed 30 complete chloroplast genomes with sequence lengths ranging from 151,655 bp to 153,235 bp before alignment and 157,060 bp after alignment correction, containing 6,417 variable sites and 148,565 conserved sites.

**Figure 5.**
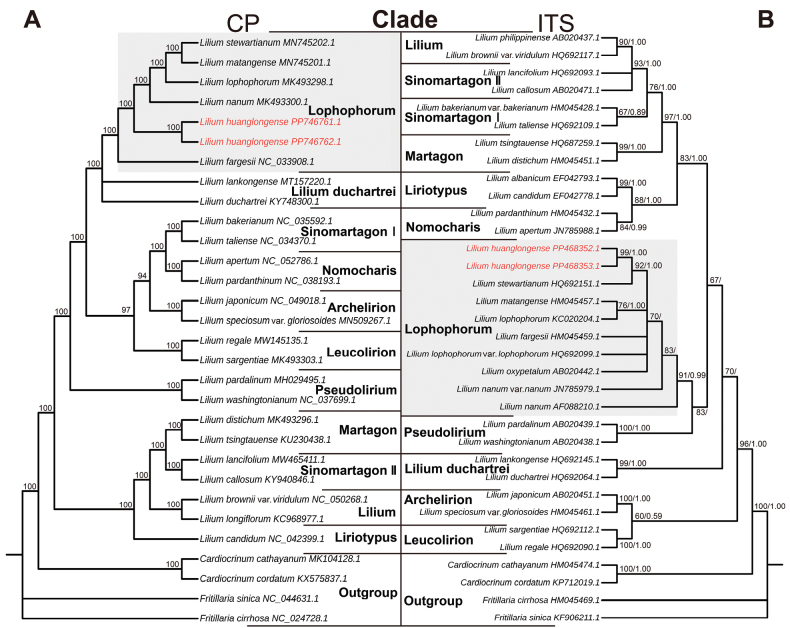
Maximum Likelihood (ML) phylogenetic analysis of selected species of *Lilium* based on **A** complete plastome DNA and **B** nuclear ITS sequence. Numbers at nodes indicate bootstrap percentages (BS) for ML. In **B**, the values to the left of the “/” represent the bootstrap support (BS), while those to the right indicate the Bayesian posterior probability (PP).

The phylogenetic analysis indicates that the Lophophorum-clade is monophyletic, supported by both chloroplast and ITS phylogenies, with support values of 100% (Fig. 5A) and 91%/0.99 (Fig. 5B, Suppl. material 2), respectively. These results are consistent with previous works (Gao et al. 2013a; Yuan and Gao 2024). In the plastid phylogeny, *L.fargesii* is resolved as sister to all other species within the Lophophorum-clade. Within this successively branching clade, both individuals of *L.huanglongense* form a monophyletic group, which is sister to *L.nanum*, *L.lophophorum*, *L.matangense* and *L.stewartianum*. In the ITS phylogeny, *L.huanglongense* is sister to *L.stewartianum*, whereas in the plastid phylogeny, *L.stewartianum* forms a clade with *L.matangense* (Fig. 5).

### ﻿Taxonomic treatment

#### ﻿ Lilium huanglongense

Taxon classificationPlantaeLilialesLiliaceae

T.Wang & Y.D.Gao sp. nov.

6EC5D120-AE0F-5174-BFE0-D091DE594AD5

urn:lsid:ipni.org:names:77356314-1

**Figure 6.**
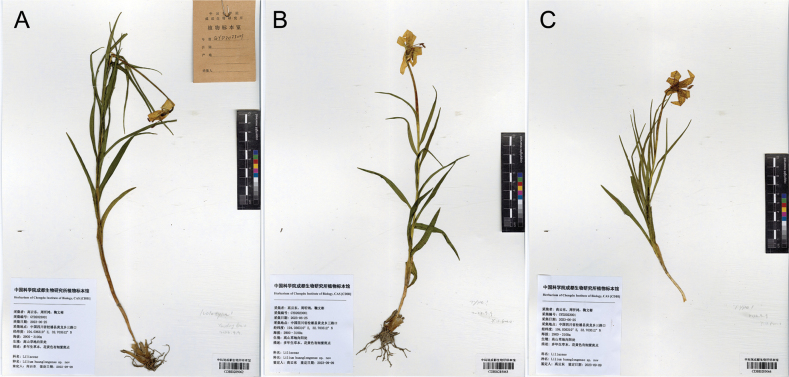
Type Specimens of *Liliumhuanglongense* T.Wang & Y.D.Gao, sp. nov. **A** holotype CDBI0285062 **B** type CDBI0285063 **C** type CDBI0285064.

Figs 1 , 2 , 3 , 4 , 6 , Table 1 , Suppl. material 1: table S1

##### Type.

China • Sichuan: Songpan County, Huanglong National Natural Reserve, 30 June 2023, *Y.D. Gao GYD2023001* (holotype: CDBI 0285062) (Fig. 6).

##### Diagnosis.

*Liliumhuanglongense* is most similar to *L.fargesii* and *L.stewartianum*, but can be distinguished from *L.fargesii* by its yellow tepals and stamens that are longer than the pistil and, in contrast to *L.stewartianum*, *L.huanglongense* lacks a deep, slender floral tubes (the height of the cone formed by the connivent tepals) and has a trilobed, non-inflated stigma. (Table 1, Suppl. material 1: table S1)

##### Description.

Bulb ca. 1.2–1.5 cm in diam., ovoid; scales 1.5–3 × ca. 8 mm, lanceolate, yellow. Stem 15–40 cm long, smooth, basal part red, red colour gradually fading and becoming green with reddish-brown speckles towards the apex of stem. Leaves 5.0–12.0 × 0.3–0.7 cm, scattered, mostly in middle and distal parts of stem, linear, margin recurved, smooth. Flowers actinomorphic, solitary, ca. 4–5 cm in diameter, nodding, Tepals 3.0–3.5 × 0.7–1.0 cm, lanceolate, margin revolute, yellow, with scattered, purple or purplish-brown spots mainly concentrated in the basal part; inner tepal nectaries with cristate projections on both surfaces, green; outer ones glabrous, with a green glistening nectarial channel at the base. Filaments 2–2.5 cm, glabrous; anthers 7–9 × ca. 2 mm, narrowly oblong, brown. Ovary 0.8–1 × ca. 0.3 cm, cylindrical. Style 0.8–1.2 cm, shorter than filaments, three-lobed without inflation, curved upwards. Capsule ca. 2 × 1.5 cm, oblong.

##### Phenology.

Flowering from June to July.

##### Habitat and distribution.

Occurring in alpine meadows on limestone slopes near streams, at altitudes of 3000–3300 m. This species is only known from three locations (one destroyed) in Huanglong National Nature Reserve, Songpan, NW Sichuan.

##### Etymology.

The epithet adopted here is derived from Huanglong National Natural Reserve, the site of discovery of this species.

##### Conservation status and IUCN preliminary assessment.

We conducted surveys in collaboration with staff from the Huanglong National Nature Reserve in Sichuan Province, China, covering approximately 150 km^2^. The species *L.huanglongense* was found at only three locations. The Extent of Occurrence (EOO) for this species was calculated to be approximately 5.361 km^2^, while its Area of Occupancy (AOO) was estimated at around 0.509 km^2^. During our field surveys, we observed that the species’ habitat is highly unstable due to annual summer floods and rockfalls. In the summer of 2023, one of the previously known sites was completely destroyed by a mudslide, resulting in the loss of all individuals at that location (Fig. 4A). At the remaining two locations, we recorded approximately 30 mature individuals in total each year. Given the limited distribution, small population size and the instability of its habitat, we propose that *L.huanglongense* be classified as Critically Endangered (CR, B1i+ii, C1) according to IUCN Red List Criteria (IUCN 2024).

## ﻿Discussion

Previously, we documented the presence of two distinct flower morphologies within the Lophophorum-clade, which may reflect parallel evolution as lilies rapidly adapt to diverse environments (Gao et al. 2015; Yuan and Gao 2024). This suggests that the former classification of *Lilium* into subgenera, based solely on floral morphological differences, may not be entirely valid (Watanabe et al. 2021). The Lophophorum-clade further supports the notion that parallel evolution is prevalent within the genus *Lilium* (Gao et al. 2015; Yuan and Gao 2024). Consequently, caution is warranted when assessing subgeneric affinities, based exclusively on morphological characteristics in this genus.

Molecular phylogenetic analysis demonstrated that *Liliumhuanglongense* occupies a distinct position within the Lophophorum-clade. The Maximum Likelihood (ML) tree, based on chloroplast data, shows *L.huanglongense* as sister to *L.nanum*, *L.lophophorum*, *L.matangense* and *L.stewartianum* (Fig. 5A). In contrast, both the ML and Bayesian Inference (BI) trees, based on ITS data, place *L.huanglongense* and *L.stewartianum* in a monophyletic group (Fig. 5B), revealing a discordance between nuclear and plastid phylogenies (Fig. 5). This discrepancy may result from incomplete lineage sorting (ILS) and introgression. However, our previous studies suggest that plastid phylogeny better reflects the geographic relationships amongst species, with introgression being a more plausible explanation (Gao et al. 2013b, 2015).

Within the plastid genome tree, *Liliumfargesii* occupies the earliest diverging position within the *Lophophorum clade*-clade (Fig. 5A). The emergence of *L.huanglongense* may have occurred during times of environmental fluctuation, such as the Quaternary Ice Age, followed by subsequent environmental isolation from its potential ancestral species during interglacial periods (Davis et al. 2005). This aligns with the observed distribution pattern, indicating that *L.huanglongense* is confined to a unique geographic position within the entire clade (Fig. 1), similar to other tepal-recurved members (e.g. *L.matangense*, *L.stewartianum*, Fig. 1) that are sporadically distributed in the Hengduan Mountains region. The limited population size and few populations of these tepal-recurved members of the Lophophorum clade clade suggest that a widely-distributed common ancestor may have existed previously, with the current relic pattern resulting from long-term isolation.

Morphologically, *Liliumhuanglongense* is characterised by its compact stature, pale yellow perianth (Fig. 2A), flattened and delicate floral structure and dense basal foliage, distinguishing it from morphologically similar species. The pale yellow perianth of *L.huanglongense* sets it apart from *L.matangense* (Fig. 2C) and *L.fargesii* (Fig. 2D), which typically exhibit flowers with perianth colours ranging from white to green. Additionally, its relatively small size and flattened floral structure (short tube length) differentiate it from *L.stewartianum* (Fig. 2E), which features a deep, slender floral funnel (the height of the cone formed by the connivent tepals). Our comparisons also revealed significant differences in the proportions of floral organs amongst these species. *Liliumhuanglongense* has flattened floral parts, with the flowers and stamens nearly in the same plane (Fig. 2A). In contrast, the pistils and stamens of similar species, such as *L.fargesii*, *L.stewartianum* and *L.matangense*, clearly protrude from the perianth.

The specialised perigone structure in *L.huanglongense* is likely the result of localised plant-environment interactions, particularly with its pollinators. These pollinators play a crucial role in driving morphological evolution (Van der Niet and Johnson 2012; Van der Niet et al. 2014) and have influenced the delicate floral features of *L.huanglongense*, such as its pale yellow perianth and flattened floral structure, which may have evolved to attract specific pollinators in its habitat. *Liliumhuanglongense* exhibits a shorter style than *L.fargesii* and a shorter floral tube (the height of the cone formed by the connivent tepals) than *L.stewartianum*. These differences in floral morphology may have evolved in response to varying pollinators, indicating that pollination syndrome may play a key role in the speciation process of this new species.

Geographically, *Liliumhuanglongense* bridges the distribution gap between *L.fargesii*, native to central China and other species inhabiting the south-western alpine mountains (Fig. 1). The entire Lophophorum-clade extends over a broad geographic range from Hunan (*L.fargesii*) in the east to the western Himalayas (*L.nanum*) in the west. This distribution encompasses mesic broadleaf forests in the central Daba Mountain system, alpine scrublands in the south-western Hengduan Mountains and extends to the alpine scrub meadows of the Himalayas (Gao et al. 2013b). The divergence of the Lophophorum clade clade is likely due to historical geological and climatic changes in the Qinling-Dabashan-Hengduan-Himalayan region (Gao et al. 2013b; Xing and Ree 2017). This region, characterised by a series of mountain ranges and diverse ecological niches, spans central and south-western China and supports substantial biodiversity.

In conclusion, *Liliumhuanglongense* is a morphologically and molecularly distinct new species within the Lophophorum-clade. This discovery not only contributes to the diversity of the genus, but also fills a geographical gap west of the Qinling Mountains, at the confluence of the Hengduan Mountains. However, our understanding of the Lophophorum-clade remains incomplete. To enhance our comprehension of its phylogenetic relationships and gain a comprehensive understanding of the biogeographic processes involved, further literature reviews, fieldwork and additional collection of morphological and molecular data are necessary.

## Supplementary Material

XML Treatment for Lilium huanglongense

## ﻿Additional information

### Author ORCIDs

Ting Wang https://orcid.org/0009-0005-2338-7011

Yumei Yuan https://orcid.org/0000-0002-4312-5167

Ting-Hong Zhou https://orcid.org/0000-0002-0647-478X

Yundong Gao https://orcid.org/0000-0002-0534-2128

### Data availability

All of the data that support the findings of this study are available in the main text or Supplementary Information.

## Supplementary materials

Supplementary material 1 Supplementary information Data type xlsx Explanation note **table S1.** Comparison of measurements and morphology of specimens of *Liliumhuanglongense*, *Liliumstewartianum*, *Liliumfargesii* and *Liliummatangense*. **table S2.** GenBank accession numbers for sequences utilised in phylogenetic analyses. https://binary.pensoft.net/file/1241943This dataset is made available under the Open Database License (http://opendatacommons.org/licenses/odbl/1.0/). The Open Database License (ODbL) is a license agreement intended to allow users to freely share, modify, and use this Dataset while maintaining this same freedom for others, provided that the original source and author(s) are credited.Ting Wang, Yumei Yuan, Ting-Hong Zhou, Yundong Gao

Supplementary material 2 Phylogenetic tree constructed from 34 ITS sequences using Bayesian analysis Data type pdf https://binary.pensoft.net/file/1241944This dataset is made available under the Open Database License (http://opendatacommons.org/licenses/odbl/1.0/). The Open Database License (ODbL) is a license agreement intended to allow users to freely share, modify, and use this Dataset while maintaining this same freedom for others, provided that the original source and author(s) are credited.Ting Wang, Yumei Yuan, Ting-Hong Zhou, Yundong Gao

## References

[B1] Allen GC Flores-Vergara MA Krasynanski S Kumar S Thompson WF (2006) A modified protocol for rapid DNA isolation from plant tissues using cetyltrimethylammonium bromide. Nature Protocols 1(5): 2320–2325. 10.1038/nprot.2006.384 17406474

[B2] Bachman SP Moat J Hill A de la Torre J Scott B (2011) Supporting Red List threat assessments with GeoCAT: Geospatial conservation assessment tool. ZooKeys 150: 117–126. 10.3897/zookeys.150.2109 PMC3234434 22207809

[B3] Baker JG (1874) Revisions of the genera and species of Tulipeae. Botanical Journal of the Linnean Society 14(76): 211–310. 10.1111/j.1095-8339.1874.tb00314.x

[B4] Smith WW (1923) Notes on Chinese Lilies. Transactions of the Botanical Society of Edinburgh 28(1–4): 122–160. 10.1080/03746602309469374

[B5] Chen S Zhou Y Chen Y Gu J (2018) fastp: An ultra-fast all-in-one FASTQ preprocessor. Bioinformatics 34(17): i884–i890. 10.1093/bioinformatics/bty560 PMC6129281 30423086

[B6] Davis MB Shaw RG Etterson JR (2005) Evolutionary responses to changing climate. Ecology 86(7): 1704–1714. 10.1890/03-0788

[B7] Franchet AR (1892) Les lis de la Chine et du Thibet. Journal de Botanique (Morot) 6(18): 304–321.

[B8] Franchet AR (1898) Plantarum sinensium ecloge secunda. Journal de Botanique (Morot) 12: 177–196. 10.1080/00378941.1898.10830840

[B9] Gao YD Gao XF (2016) Accommodating *Nomocharis* in *Lilium* (Liliaceae). Phytotaxa 277(2): 205–210. 10.11646/phytotaxa.277.2.8

[B10] Gao YD Hohenegger M Harris AJ Zhou SD He XJ Wan J (2012) A new species in the genus *Nomocharis* Franchet (Liliaceae): Evidence that brings the genus *Nomocharis* into *Lilium*. Plant Systematics and Evolution 298(1): 69–85. 10.1007/s00606-011-0524-1

[B11] Gao YD Zhou SD He XJ (2013a) *Liliumyapingense* (Liliaceae), a New Species from Yunnan, China, and its Systematic Significance Relative to *Nomocharis*. Annales Botanici Fennici 50(3): 187–194. 10.5735/085.050.0311

[B12] Gao YD Harris AJ Zhou SD He XJ (2013b) Evolutionary events in *Lilium* (including *Nomocharis*, Liliaceae) are temporally correlated with orogenies of the Q–T plateau and the Hengduan Mountains. Molecular Phylogenetics and Evolution 68(3): 443–460. 10.1016/j.ympev.2013.04.026 23665039

[B13] Gao YD Harris AJ He XJ (2015) Morphological and ecological divergence of *Lilium* and *Nomocharis* within the Hengduan Mountains and Qinghai-Tibetan Plateau may result from habitat specialization and hybridization. BMC Evolutionary Biology 15(1): 147. 10.1186/s12862-015-0405-2 26219287 PMC4518642

[B14] IUCN (2024) Guidelines for Using the IUCN Red List Categories and Criteria. Version 16. Prepared by the Standards and Petitions Committee. http://www.iucnredlist.org/documents/RedListGuidelines.pdf [Accessed 20 June 2024]

[B15] Jin JJ Yu WB Yang JB Song Y dePamphilis CW Yi TS Li DZ (2020) GetOrganelle: A fast and versatile toolkit for accurate de novo assembly of organelle genomes. Genome Biology 21(1): 241. 10.1186/s13059-020-02154-5 32912315 PMC7488116

[B16] Kalyaanamoorthy S Minh BQ Wong TK Von Haeseler A Jermiin LS (2017) ModelFinder: Fast model selection for accurate phylogenetic estimates. Nature Methods 14(6): 587–589. 10.1038/nmeth.4285 28481363 PMC5453245

[B17] Katoh K Standley DM (2013) MAFFT multiple sequence alignment software version 7: Improvements in performance and usability. Molecular Biology and Evolution 30(4): 772–780. 10.1093/molbev/mst010 23329690 PMC3603318

[B18] Klotzsch F Garcke A (1862) Die botanischen ergebnisse der reise Seiner Königl. Hoheit des prinzen Waldemar von Prussen in den jahren 1845 und 1846. Verlag der Königlichen Geheimen oberhofbuchdruckerei (R. Becker), Berlin, 53 pp.

[B19] Lemoine F Correia D Lefort V Doppelt-Azeroual O Mareuil F Cohen-Boulakia S Gascuel O (2019) NGPhylogeny. fr: New generation phylogenetic services for non-specialists. Nucleic Acids Research 47(W1): W260–W265. 10.1093/nar/gkz303 PMC6602494 31028399

[B20] Letunic I Bork P (2024) Interactive Tree of Life (iTOL) v6: Recent updates to the phylogenetic tree display and annotation tool. Nucleic Acids Research 52(W1): W78–W82. 10.1093/nar/gkae268 PMC11223838 38613393

[B21] Liang SY (1995) Chorology of Liliaceae (*s. str.*) and its bearing on the Chinese flora. Journal of Systematics and Evolution 33(1): 27–51.

[B22] Liang SY Tamura M (2000) *Lilium* L. In: Wu ZY Raven PH (Eds) Flora of China Vol.24. Science Press, Beijing; & Missouri Botanical Garden Press, St. Louis, 135–159.

[B23] Liu L Wang Q Zhang Z He X Yu Y (2023) MATO: An updated tool for capturing and analyzing cytotaxonomic and morphological data. The Innovation Life 1(1): 100010. 10.59717/j.xinn-life.2023.100010

[B24] Peruzzi L (2016) A new infrafamilial taxonomic setting for Liliaceae, with a key to genera and tribes. Plant Biosystems - An International Journal Dealing with all Aspects of Plant Biology 150: 1341–1347. 10.1080/11263504.2015.1115435

[B25] POWO (2024) Plants of the World Online. Facilitated by the Royal Botanic Gardens, Kew. http://www.plantsoftheworldonline.org/ [Accessed 20 June 2024]

[B26] Ronquist F Teslenko M Van Der Mark P Ayres DL Darling A Höhna S Larget B Liu L Suchard MA Huelsenbeck JP (2012) MrBayes 3.2: Efficient Bayesian phylogenetic inference and model choice across a large model space. Systematic Biology 61(3): 539–542. 10.1093/sysbio/sys029 22357727 PMC3329765

[B27] Talavera G Castresana J (2007) Improvement of phylogenies after removing divergent and ambiguously aligned blocks from protein sequence alignments. Systematic Biology 56(4): 564–577. 10.1080/10635150701472164 17654362

[B28] Tamura K Stecher G Kumar S (2021) MEGA11: Molecular Evolutionary Genetics Analysis Version 11. In: Battistuzzi FU (Ed.) Molecular Biology and Evolution 38: 3022–3027. 10.1093/molbev/msab120 33892491 PMC8233496

[B29] Thiers B (2024) Index Herbariorum: A global directory of public herbaria and associated staff. New York Botanical Garden’s Virtual Herbarium. http://sweetgum.nybg.org/ih/ [Accessed 29 September 2024]

[B30] Van der Niet T Johnson SD (2012) Phylogenetic evidence for pollinator-driven diversification of angiosperms. Trends in Ecology & Evolution 27(6): 353–361. 10.1016/j.tree.2012.02.002 22445687

[B31] Van der Niet T Peakall R Johnson SD (2014) Pollinator-driven ecological speciation in plants: New evidence and future perspectives. Annals of Botany 113(2): 199–212. 10.1093/aob/mct290 24418954 PMC3890394

[B32] Watanabe ST Hayashi K Arakawa K Fuse S Nagamasu H Ikeda H Kuyama A Suksathan P Poopath M Pooma R (2021) Biosystematic studies on *Lilium* (Liliaceae) I. Phylogenetic analysis based on chloroplast and nuclear DNA sequences and a revised infrageneric classification. Acta Phytotaxonomica et Geobotanica 72(3): 179–204.

[B33] Xing Y Ree RH (2017) Uplift-driven diversification in the Hengduan Mountains, a temperate biodiversity hotspot. Proceedings of the National Academy of Sciences 114(17): E3444–E3451. 10.1073/pnas.1616063114 PMC5410793 28373546

[B34] Xu JM (1985) New species of Liliaceae from Sichuan. Journal of Systematics and Evolution 23(3): 233–234.

[B35] Yuan YM Gao YD (2024) *Liliumliangiae*, a new species in the genus *Lilium* (Liliaceae) that reveals parallel evolution within morphology. Frontiers in Plant Science 15: 1371237. 10.3389/fpls.2024.1371237 PMC11004424 38601309

[B36] Zhang D Gao F Jakovlić I Zou H Zhang J Li WX Wang GT (2020) PhyloSuite: An integrated and scalable desktop platform for streamlined molecular sequence data management and evolutionary phylogenetics studies. Molecular Ecology Resources 20(1): 348–355. 10.1111/1755-0998.13096 31599058

