# Supplementary figures and images for: ﻿Liliumhuanglongense (Liliaceae): a newly-discovered species in north-western Sichuan, China
Source: PhytoKeys. 2025 Feb 4;252:9–24. doi: 10.3897/phytokeys.252.135155 (PMC11815331; doi:10.3897/phytokeys.252.135155)

ITS BI

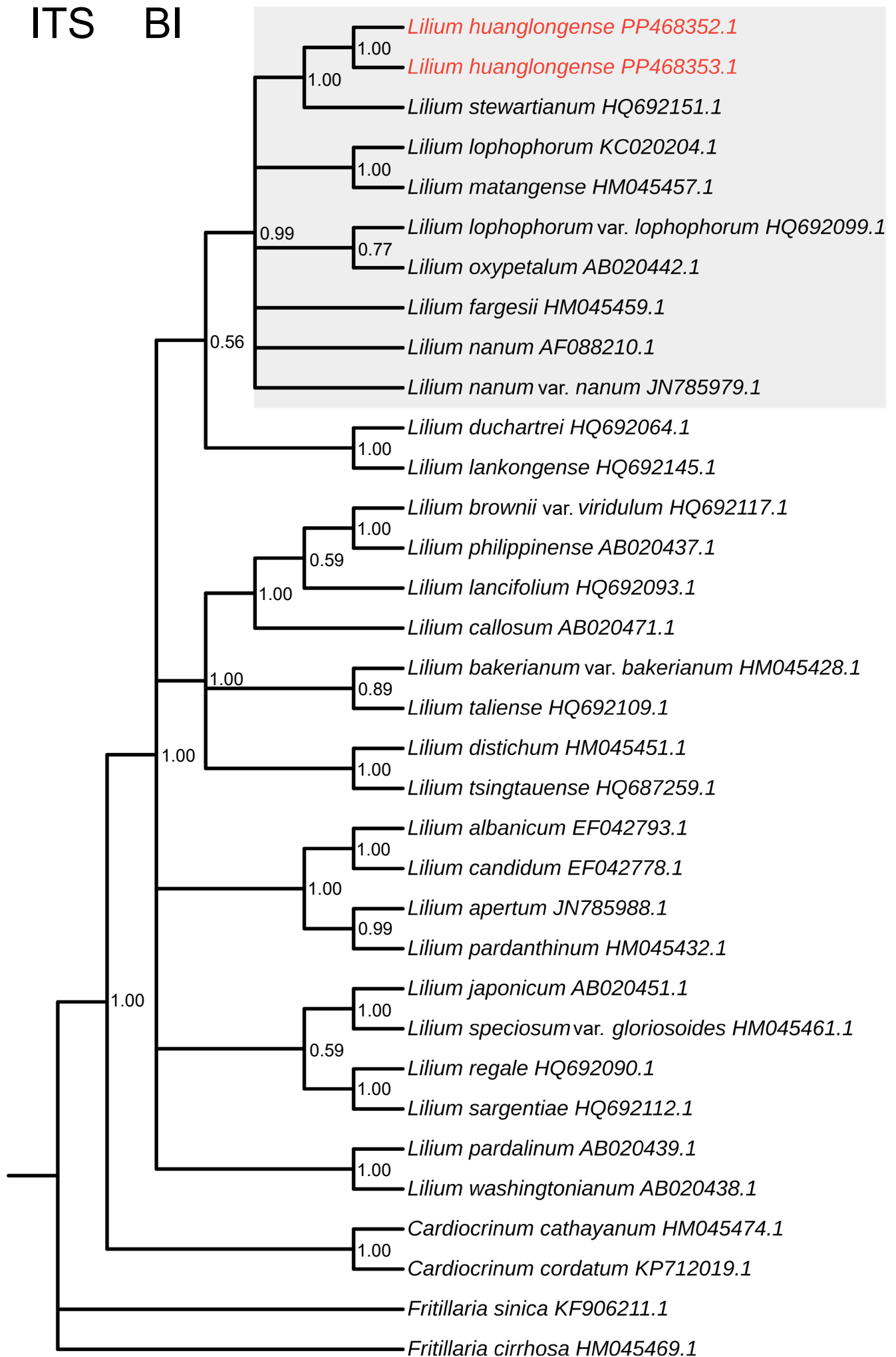

Supplement: Supplementary material 2 — Phylogenetic tree constructed from 34 ITS sequences using Bayesian analysis [file phytokeys-252-009_article-135155__-s002.pdf]
